# Comparative Study of Regulatory Circuits in Two Sea Urchin Species Reveals Tight Control of Timing and High Conservation of Expression Dynamics

**DOI:** 10.1371/journal.pgen.1005435

**Published:** 2015-07-31

**Authors:** Tsvia Gildor, Smadar Ben-Tabou de-Leon

**Affiliations:** Department of Marine Biology, Leon H. Charney School of Marine Sciences, University of Haifa, Haifa, Israel; Duke University, UNITED STATES

## Abstract

Accurate temporal control of gene expression is essential for normal development and must be robust to natural genetic and environmental variation. Studying gene expression variation within and between related species can delineate the level of expression variability that development can tolerate. Here we exploit the comprehensive model of sea urchin gene regulatory networks and generate high-density expression profiles of key regulatory genes of the Mediterranean sea urchin, *Paracentrotus lividus* (*Pl*). The high resolution of our studies reveals highly reproducible gene initiation times that have lower variation than those of maximal mRNA levels between different individuals of the same species. This observation supports a threshold behavior of gene activation that is less sensitive to input concentrations. We then compare Mediterranean sea urchin gene expression profiles to those of its Pacific Ocean relative, *Strongylocentrotus purpuratus* (*Sp*). These species shared a common ancestor about 40 million years ago and show highly similar embryonic morphologies. Our comparative analyses of five regulatory circuits operating in different embryonic territories reveal a high conservation of the temporal order of gene activation but also some cases of divergence. A linear ratio of 1.3-fold between gene initiation times in *Pl* and *Sp* is partially explained by scaling of the developmental rates with temperature. Scaling the developmental rates according to the estimated *Sp-Pl* ratio and normalizing the expression levels reveals a striking conservation of relative dynamics of gene expression between the species. Overall, our findings demonstrate the ability of biological developmental systems to tightly control the timing of gene activation and relative dynamics and overcome expression noise induced by genetic variation and growth conditions.

## Author Summary

Embryonic development necessitates a delicate balancing act. On one hand, precise regulation of the expression of developmental genes is crucial for the maintenance of morphology and function. On the other hand, these same regulatory networks must allow normal development to proceed through genetic variation and environmental changes. To learn how regulatory circuits operate robustly within natural variation, we study the temporal expression profiles of key regulatory genes in the Mediterranean sea urchin, *Paracentrotus lividus*, and compare them to those of its Pacific Ocean relative, *Strongylocentrotus purpuratus*. These species shared a common ancestor about 40 million years ago and show highly similar embryonic morphologies. Our studies reveal highly reproducible gene initiation times that show lower variations than the variations in maximal mRNA levels within the species (*Pl*). We observe high interspecies conservation of the temporal order of gene activation within regulatory circuits and some cases of divergence. This conservation was even more profound when expression levels were normalized and scaled to the different developmental rates between the species. Our findings highlight that, despite genetic variations and different growth conditions, expression dynamics in developmental gene regulatory networks are extremely conserved over 40 million years of evolution.

## Introduction

Normal development requires precise temporal control of differential gene expression, yet development must be robust to natural genetic variation and environmental changes. [1–3]. This resilience of developmental systems is important for keeping a wide genotypic pool adaptable in changing environmental conditions and thus, for the survival of the species [4,5]. Identifying how the control systems overcome genetic and environmental changes is important to the mechanistic understanding of developmental processes and their evolution [1,3,4]. Specifically, comparing different aspects of expression dynamics between individuals within the species and between closely related species can illuminate the range of variation in temporal expression that can still produce similar embryonic structures [1,6–8].

Comparative studies of interspecies differences in the kinetics of gene regulatory circuits can provide predictions for *trans* and *cis* evolutionary changes in circuit connectivity. The timing of gene expression depends on the temporal expression profiles of the inputs (*trans*) and the logic applied on the inputs by the *cis*-regulatory modules [9,10] (S1A–S1C Fig). For example, if two inputs are activated sequentially and the target *cis*-regulatory element requires both of them (necessary inputs, AND logic), the target gene will turn on only after the activation of the later input gene (S1B Fig) [9]. If the two inputs are additive (OR logic), the target gene will turn on immediately after the activation of the earlier input gene [11] (S1C Fig). Thus, evolutionary changes in *cis*-regulatory logic, *e*.*g*. from AND to OR, could result in changes in gene expression timing. Comparing the expression profiles of both input and target genes between two species can provide predictions for changes in input dynamics and in the target's *cis*-regulatory logic.

**Fig 1:**
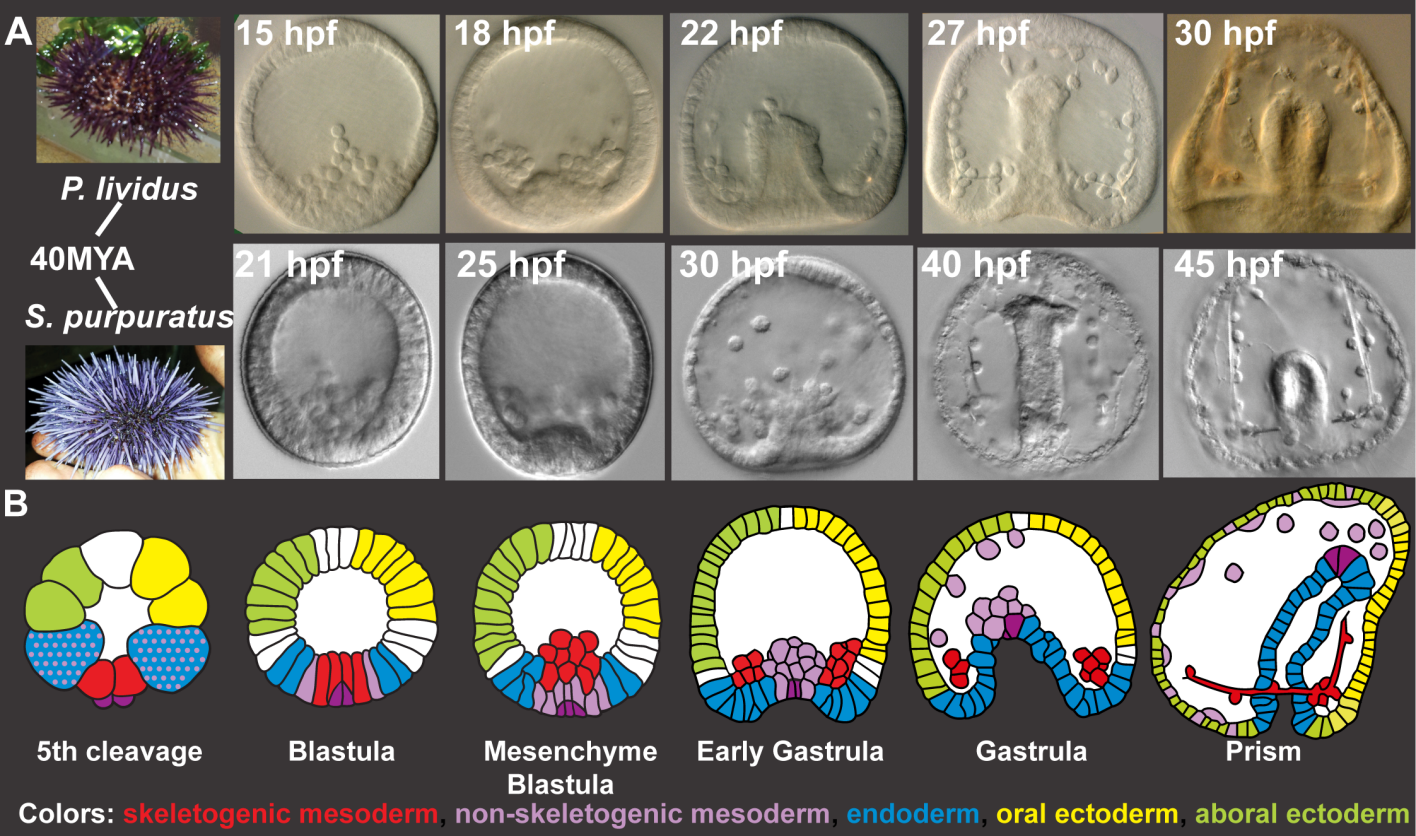
*Sp* and *Pl* development. **A,** Comparison of *Pl* (top) and *Sp* (bottom) embryo development up to prism stage. **B,** Schematic diagrams of sea urchin cell lineages [28]. Red–skeletogenic mesoderm, light purple–non skeletogenic mesoderm, blue–endoderm, yellow–oral ectoderm, green–aboral ectoderm, dark purple–small micromeres.

Comparative studies of temporal variation of gene regulatory circuits between related species must rely on detailed experimentally-based models of the gene regulatory networks in these organisms. The current models of the gene regulatory networks that drive ectoderm, endoderm and mesoderm specification in the sea urchin embryo are among the most comprehensive of their kind and are based on experimental studies in a few main species. [12–16]. The purple sea urchin, *Strongylocentrotus purpuratus* (*Sp*) inhabits the Pacific coasts of North America while the sea urchin *Paracentrotus lividus* (*Pl*) inhabits the eastern Atlantic Ocean and the Mediterranean Sea. These species shared a common ancestor about 40 million years ago and the average similarity in their coding sequences is about 85%, which is similar to that found between human and mouse. The growth temperature of these two species is different, reflecting their different environments; *Pl* embryos will successfully develop over a temperature range that is higher than *Sp* (standard lab temperatures 18°C versus 15°C, respectively). These species show apparent similarities in size, morphology, spatial gene expression patterns and gene regulatory networks, despite their genomic divergence and geographic distance (Fig 1A) [14–25]. High resolution studies of the temporal expression profiles of more than a hundred regulatory and differentiation genes that operate at different embryonic territories were performed for *Sp* [13,26], but equivalent information for *Pl* is still limited [18].

Here, we perform high-resolution quantitative analysis of the transcriptional expression profiles of key regulatory genes in *Pl*, asses the temporal expression variation within the species and compare gene expression dynamics to those measured in *Sp* [26]. For these studies, we selected regulatory circuits that operate in five embryonic territories and contain common network motifs found in many other gene regulatory networks, such as positive feedback and feedforward structures. The positive feedback circuitry locks down a specification state within a cell (intracellular, S1D Fig) or within an embryonic territory (intercellular, S1E Fig) and is important for cell fate decision [15,27–29]. Coherent and incoherent feedforward motifs are used for the sequential activation of genes in a cell (S1F Fig) [30–32]. Our results portray a tight control of timing of gene activation that is highly conserved between the species despite their genetic and geographic distance. The developmental rates of the two species scale linearly, in agreement with the species’ different growth temperatures. When we scale the developmental rates of the two species, we reveal a remarkable conservation of relative expression dynamics. Thus our study illuminates the dynamic properties of biological regulatory systems and their ability to control relative dynamics accurately despite genetic and growth condition differences.

## Results

### High resolution temporal profiles of *Paracentrotus lividus* regulatory genes reveal tight control of initiation timing

#### Generation of temporal expression profiles of 25 developmental genes in early development of *Paracentrotus lividus*

The models of the sea urchin embryo gene regulatory networks describe cell fate specification and differentiation up to gastrulation [12,15,16,33,34]. During this time interval, multiple developmental programs are executed in different embryonic territories (Fig 1A and 1B). Relevant to our studies are the large micromeres that differentiate to skeletogenic mesoderm and generate the larval skeleton (Fig 1B). The ring of cells adjacent to the micromeres at early blastula stage is the non-skeletogenic mesoderm that gives rise to several cell lineages, including pigment cells. Gastrulation begins with the invagination of the endodermal cells and the formation of a gut. In the ectoderm, we focus on the oral ectoderm where the mouth forms and the aboral ectoderm that differentiates into squamous epithelium. The region between the oral and aboral ectoderm is the ciliary band and the apical domain is located at its most animal region.

To generate temporal expression profiles of *Pl* embryogenesis from the fertilized egg to prism stage, we pooled thousands of *Pl* embryos in 1–2 hour intervals up to 30 hours post fertilization (hpf), and measured gene expression levels by quantitative PCR (QPCR, see experimental methods for details). We studied 25 regulatory and differentiation genes that initiate the specification of the skeletogenic mesoderm, the aboral non-skeletogenic mesoderm that generates pigment cells, the endoderm, the oral ectoderm and the aboral ectoderm. We repeated the measurements for three pairs of parents to study the natural variation of mRNA level and dynamics between different individuals from the same species. Thus, offspring from each set of parents are considered here as a different individual/biological replicate. The temporal profiles of all genes at the three biological repeats, their averages and the standard deviations are provided in the supporting S1 Dataset.

#### Highly repeatable gene initiation timing and mild variations in maximal mRNA levels in *Paracentrotus lividus* expression profiles

**Fig 2:**
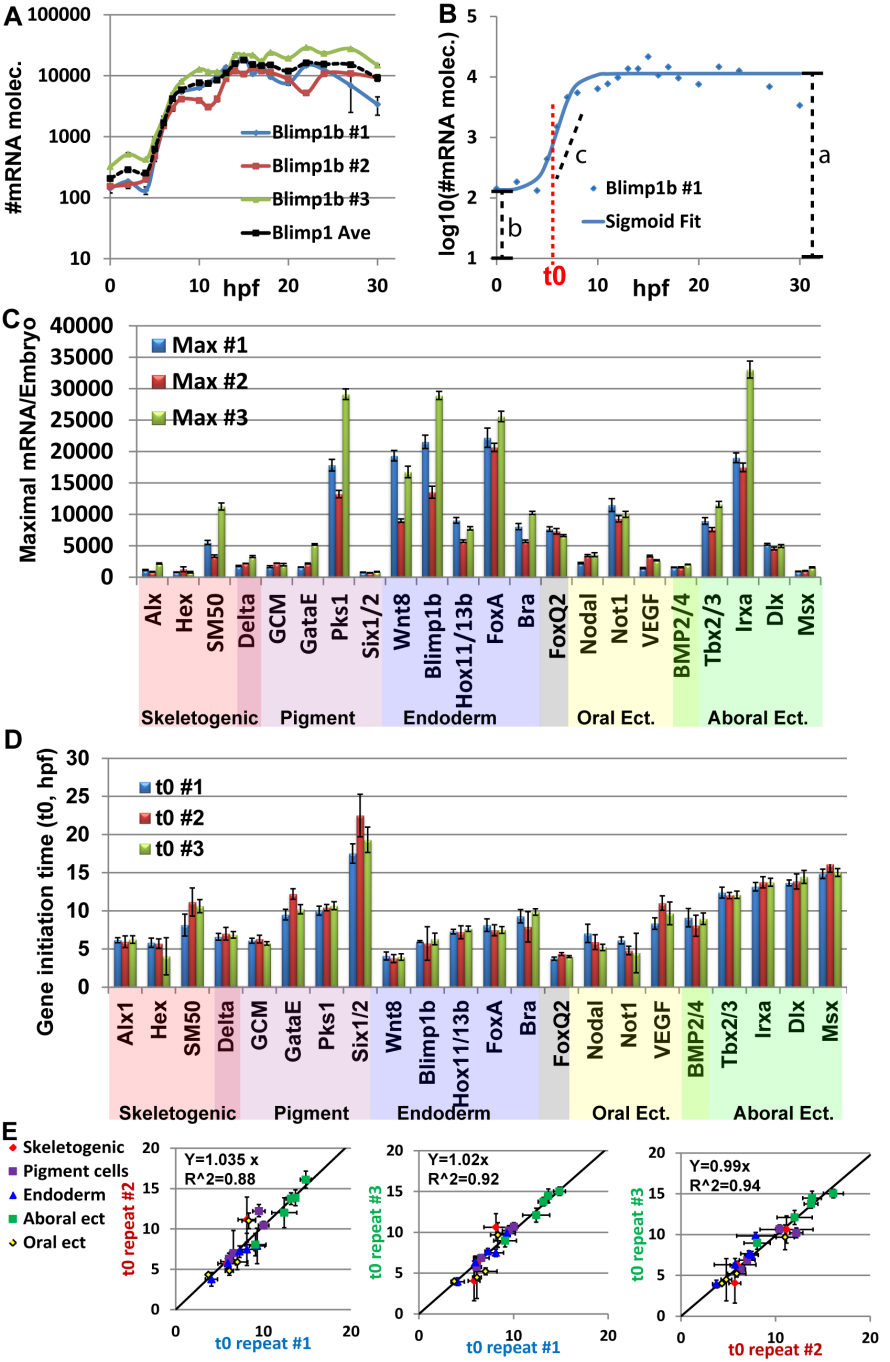
Mild variations of mRNA maximal levels and highly repeatable gene initiation times in *Pl* expression profiles. **A,** *blimp1b* mRNA level in three biological repeats (blue, red and green lines) and average of the three replicates (black dashed line). Expression is presented in Log_10_ scale. **B,** Example for the use of the sigmoid fit to measure the initiation time, t0 (See text). Blue dots are the measured expression of *blimp1b* at #1 biological repeat, blue line is the sigmoidal function using the fit parameters obtained from Matlab. Red dashed line indicates the time of half rise, t0, which is one of the parameters of the sigmoid function. Black dashed lines indicate the parameters a, b, c of the sigmoid function. **C,** Maximal mRNA level at the three biological repeats. Error bars represent technical standard error. **D,** Gene initiation time of the zygotic genes in *Pl* extracted using the sigmoid fit. Error bars represent 95% confidence bounds reported by Matlab. **E,** The ratio between estimated initiation times in pairs of biological repeats, interception was set at zero. Linear relations with slopes of ~1 indicate highly reproducible developmental rates within the species.

Our results reveal highly reproducible initiation time of gene activation between different biological replicates while the mRNA levels demonstrate higher variation for most genes. An example of our measurements for the gene *blimp1b* is presented in Fig 2A and 2B. The variations in *blimp1b* expression between the three biological repeats are the least pronounced during the initial phase of its activation (Fig 2A). This is quite surprising as the initiation time is the most dynamic phase in gene expression when the expression increases from basal to maximal levels. Therefore high variations between biological replicates could be expected at this highly dynamic period. Yet, highly repeatable gene initiation timing is observed for many of the genes (S2 Fig).

To estimate the variation in mRNA expression we measured the maximal mRNA level in each biological repeat (Fig 2C). An average of 1.5-fold difference between the maximal mRNA levels of different biological repeats was detected. We divide the standard deviation with the average maximal level of the three biological replicates to normalize the standard deviation with respect to gene expression level. The normalized standard deviation varies from 7% (*dlx*) to 64% (*gatae*) with average of 29% over all genes (Fig 2C). For most genes, the biological variations were larger than the technical variation in the measurements, indicating an actual difference in expression level between individuals. However, a 1.5-fold difference is only 0.6 cycle difference in QPCR, which is close to the QPCR resolution limit. Additionally, the Pearson correlation of maximal mRNA levels between the biological repeats is strong (0.93–0.94). Thus, these are relatively mild differences between individuals that indicate that the order of magnitude of mRNA level is well controlled for the studied regulatory and developmental genes.

To estimate gene initiation time and quantify the biological variations in this parameter, we used the sigmoid function: log(*mRNA*(*t*)) = *a* − *b*/(1 + exp(*c*(*t* − *t*0)) (Fig 2B) as was performed in Yanai *et el*, 2011 [35]. The function is a good fit for log_10_(mRNA(t)) where *mRNA(t)* is the mRNA level at time t and the parameters correspond to time of half-rise, *t0*, log_10_ of the initial expression level (*b*), log_10_ of the average maximal expression (*a*) and expression generation slope (*c*) as demonstrated for *blimp1b* in Fig 2B (see materials and methods for details). We consider t0 as a good estimate for gene initiation time. The normalized standard deviation of t0 varies from 1.6% (*tbx2/3*) to 19% (*hex*) and has average of 8% over all genes (Fig 2D). Strong Pearson correlations between the initiation times of different biological repeats (0.95–0.97) indicate a linear relation between the developmental rates of different individuals. We used linear regression to calculate the ratio between gene initiation times of different biological repeats and obtained factors of ~1-fold in the three pairwise comparisons (Fig 2E). This illustrates highly repeatable molecular developmental rates between different biological repeats that are largely uniform across different embryonic territories.

The observed difference in the variation between initiation time and mRNA level is not due to the different estimation method (maximal level measured directly from our experimental results and initiation time estimated computationally). Calculating the maximal level using the fit parameter, *a*, which gives an estimate of log10 of the maximal level, results in a similar variation in mRNA levels to those measured directly, with an average standard deviation of 35%. There are low correlations between the variation of mRNA levels and variation of gene initiation time (0.13). Thus, our measurements reveal mild variations of mRNA levels and highly reproducible initiation timing and molecular developmental rates between different individuals within the species. The observed variations in mRNA levels and in initiation timing are not dependent on each other and could indicate differences in the molecular control mechanisms of these two properties.

### Comparing *Pl* and *Sp* temporal expression profiles illuminates similarities and changes in circuits’ connectivity

**Fig 3:**
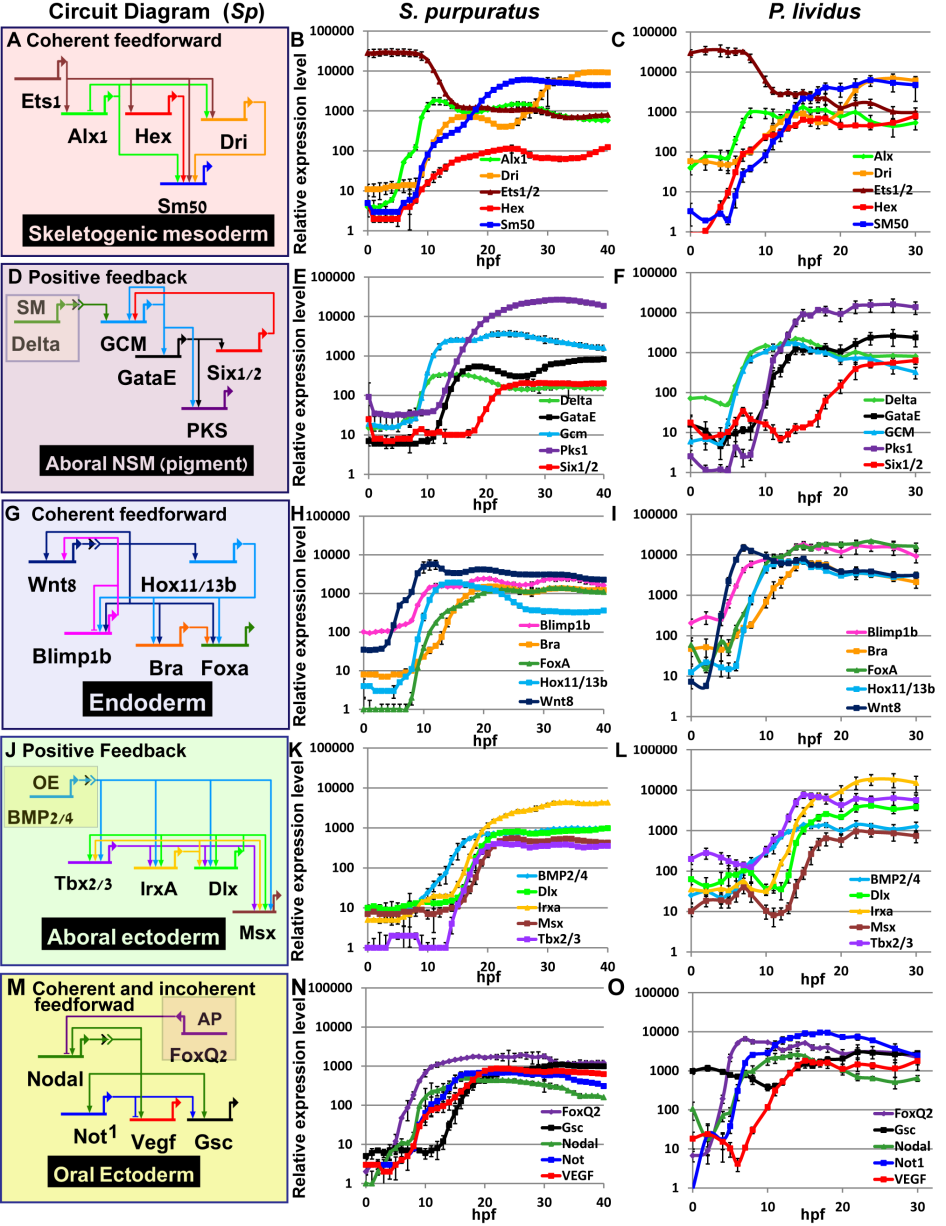
Comparison of transcriptional expression profiles of five regulatory circuits that operate at different embryonic territories. Circuit diagrams are based on experimental studies in *Sp*. **A-C**, Skeletogenic circuit: **A**, schematic diagram; **B**, time course in *Sp*; **C**, time course in *Pl*. The color code of the genes matches throughout A-C and the same is for the rest of the circuits. **D-F**, aboral non-skeletogenic mesoderm circuit (pigment cell specification), **G-I**, Endoderm specification circuit, **J-L**, aboral ectoderm circuit. **M-O**, oral ectoderm circuit. Each point in *Pl* time courses is an average of three biological repeats, see material and methods for experimental details.

Comparing gene expression profiles between *Pl* and *Sp* can identify both conserved and diverged expression patterns and suggest similarity and changes in circuits’ connectivity. High resolution time courses in *Sp* were measured by nanostring up to 48 hpf in this species [26], which includes the time interval 0–30 hpf in *Pl* (Fig 1A). While comparing actual mRNA levels between species is difficult due to the different methods used [26], comparison of initiation times and relative gene expression levels is possible. In Fig 3, we present comparative expression profiles of the studied genes separated into five regulatory circuits that initiate the specification of the skeletogenic mesoderm (Fig 3A–3C), the aboral non-skeletogenic mesoderm that form pigment cells (Fig 3D–3F), the endoderm (Fig 3G–3I), the aboral ectoderm (Fig 3J–3L) and the oral ectoderm (Fig 3M–3O). *Sp* expression profiles are taken from Materna et al, 2010, [26] (running averages of two biological replicates measured by nanostring technique, the data is available at http://vanbeneden.caltech.edu/~m/cgi-bin/hd-tc/plot.cgi). The circuit diagrams are based on experimental validations that include perturbation and *cis*-regulatory analysis in *Sp* [13,15,27,33,34,36]. Below we discuss the level of conservation of each circuit between the species in the light of our temporal expression comparison and previous studies.

#### Interspecies conservation and change of temporal profiles in the mesodermal circuits

In the skeletogenic lineage, the transcription factor Ets1 is maternal in both organisms and according to studies in *Sp* it activates the genes that encode transcription factors Alx1 and Hex. Ets1 and Alx1 are activating inputs of *dri;* and Ets1, Alx1, Hex and Dri activate the expression of the spicule matric gene *SM50* in a coherent feedforward structure (Fig 3A) [13,37,38]. In both *Sp* and *Pl*, *alx1* turns on first, and *dri* and *SM50* turn on later at a similar time (Fig 3A–3C). The similar initiation times of *dri* and *SM50* indicates that Dri is an additive but not necessary input to the activation of *SM50* (Fig 3B and 3C). The gene that encodes the transcription factor Hex turns on at the same time as *Alx1* in *Pl* but together with *dri* and *SM50* in *Sp* which might indicate a change in this gene’s regulation between the two species.

The specification of the non-skeletogenic mesoderm is initiated by the activation of the gene encoding the transcription factor GCM by Delta signaling received from the skeletogenic mesoderm (Fig 3D) [34,39–41]. According to studies in *Sp*, GCM activates *gataE* and *pks1* and feeds back to its own gene activation. GataE activates *pks1* and *six1/2*; then Six1/2 feeds back to activate *GCM* [27,34,39,42,43].The activation of *GCM* is quite rapid and *GCM* turns on immediately after *delta* expression commences in both species (Fig 3E). In both species, *gataE* and *pks1* turn on at about the same time and *six1/2* turns on later and is delayed in *Pl* compared to *Sp* (Fig 3E and 3F). Further studies are required to identify whether the observed shift in *six1/2* expression are due to *cis*-regulatory modifications or due to changes in upstream input dynamics.

#### Interspecies conservation of temporal profiles in the endodermal circuit

The endodermal circuit includes genes that have strong endodermal phenotypes, yet their spatial expression patterns are not excluded to the endoderm at all times [12,44,45]. The spatial expression of the early genes, *wnt8*, and *blimp1b* starts in the skeletogenic mesoderm and then expands to the endomesoderm cells where they are co-expressed with *hox11/13b*, *foxa* and later *bra* [12,44]. Only later (18hpf in *Sp*) the genes clear from the mesoderm and are expressed only in the endoderm [12,46,47]. After 24hpf *wnt8* is expressed in the ectoderm, *bra* and *hox11/13b* in the anterior endoderm and *foxa* and *blimp1b* in the posterior endoderm [12,44]. The links in the endoderm circuit diagram, Fig 3G, are based on extensive studies in *Sp*, executed from the onset of these genes activation and up 27hpf [12,36,44,48–51]. According to our studies, the temporal order and timing of these genes is conserved between the two species and the genes turn on at equivalent developmental times; *wnt8* turns on first and then *blimp1b* and *hox11/13b* and then *foxa* and finaly *bra* (Fig 3H and 3I). The activation of *foxa* precedes the activation of *bra* in both species, and *Spbra* is a direct input that is additive but not necessary to *Spfoxa* expression as shown in *Spfoxa cis*-regulatory analysis [36]. Our findings support a high conservation of regulatory links and *cis*-regulatory logic in the endodermal circuit between the species.

#### Interspecies variations in temporal activation in the ectoderm circuits implies *cis*-regulatory changes

Gene expression in the aboral ectoderm is boosted by the reception of BMP signaling (Fig 3J) [15,33,52,53]. BMP2/4 is expressed at the oral ectoderm but its inhibition by Chordin prevents its reception there so the activity of BMP is restricted to the aboral side of the embryo. *BMP2/4* expression is earlier in *Pl* compared to its expression in *Sp* and the expression of its target gene, *tbx2/3*, precedes the expression of its other target genes, *irxa*, *dlx* and *msx* in *Pl* while in *S*p the four genes turn on at about the same time (Fig 3K and 3L). The prominent role of Tbx2/3 in activating the aboral ectoderm regulatory genes was demonstrated in both species [15,16]. However, in *Pl*, Tbx2/3 is necessary for the activation of *msx*, *irxa* and *dl*x, implying AND logic [16] (S1B Fig), while in *Sp* Tbx2/3 and BMP activate their downstream genes in an additive manner as demonstrated by a *cis*-regulatory analysis of *dlx* [15] (OR logic, S2C Fig). Therefore, the temporal expression differences between the two species could be explained by a change in the *cis*-regulatory logic of the genes: in *Pl msx*, *dlx* and *irxa* require both Tbx2/3 and BMP2/4 inputs and thus are activated only after Tbx2/3 is on, while in *Sp* BMP2/4 is sufficient so the genes are activated earlier (see Figs 3K and 3L and S1B and S1C).

The temporal profiles of the oral ectoderm circuit show a few differences between the two species. Nodal signaling controls oral ectoderm specification [15,33,52,53] and it activates the genes that encode the transcription factors Not1 and Gsc and the ligand VEGF [14,16,20,33,53]. Nodal is restricted from the apical domain by the repressor FoxQ2 [54]. According to studies in *Sp*, Not1 activates the expression of *gsc* and represses the expression of *VEGF* in the oral ectoderm and restricts VEGF expression into two lateral domains [16,53]. According to our studies, in the two species *foxQ2* is the earliest gene and then *nodal* turns on and then Nodal’s target gene *not1* (Fig 3N and 3O). *gsc* expression is maternal and zygotic in *Pl* (Fig 3N in agreement with [16]) while in *Sp* it has only a zygotic phase (Fig 3O), [14,26]. The difference in *gsc* expression pattern could explain the weaker effect of Gsc perturbation in *Sp* [14] compared to the effect in *Pl* [16]. VEGF expression initiates together with *not1* expression in *Sp*, but only 4h after *not1* initiation in *Pl* (Fig 3N and 3O). This could indicate that VEGF is directly regulated by Nodal in *Sp*, but indirectly in *Pl*, possibly due to *cis*-regulatory changes in this gene. The delayed expression of VEGF in *Pl* means that Not1 is already active at the time of VEGF initiation and could be repressing VEGF expression in the oral ectoderm. Indeed, spatial studies of VEGF expression in *Sp* detect an early broad oral expression of VEGF [20] while at the earliest time of VEGF expression in *Pl* VEGF is restricted to two lateral domains and absent from oral ectoderm, possibly due to its repression by Not1 [55]. Thus, our high-resolution comparison of ectodermal gene expression reveals a few modifications in the timing of gene activation that could be explained in *cis*-regulatory module changes in a few of the downstream ectodermal genes.

### Temporal and level scaling of *Pl* and *Sp* expression profiles reveals strong conservation of relative expression dynamics

#### The developmental rate of the *Pl* embryo is about ×1.3 times that of *Sp.*

**Fig 4:**
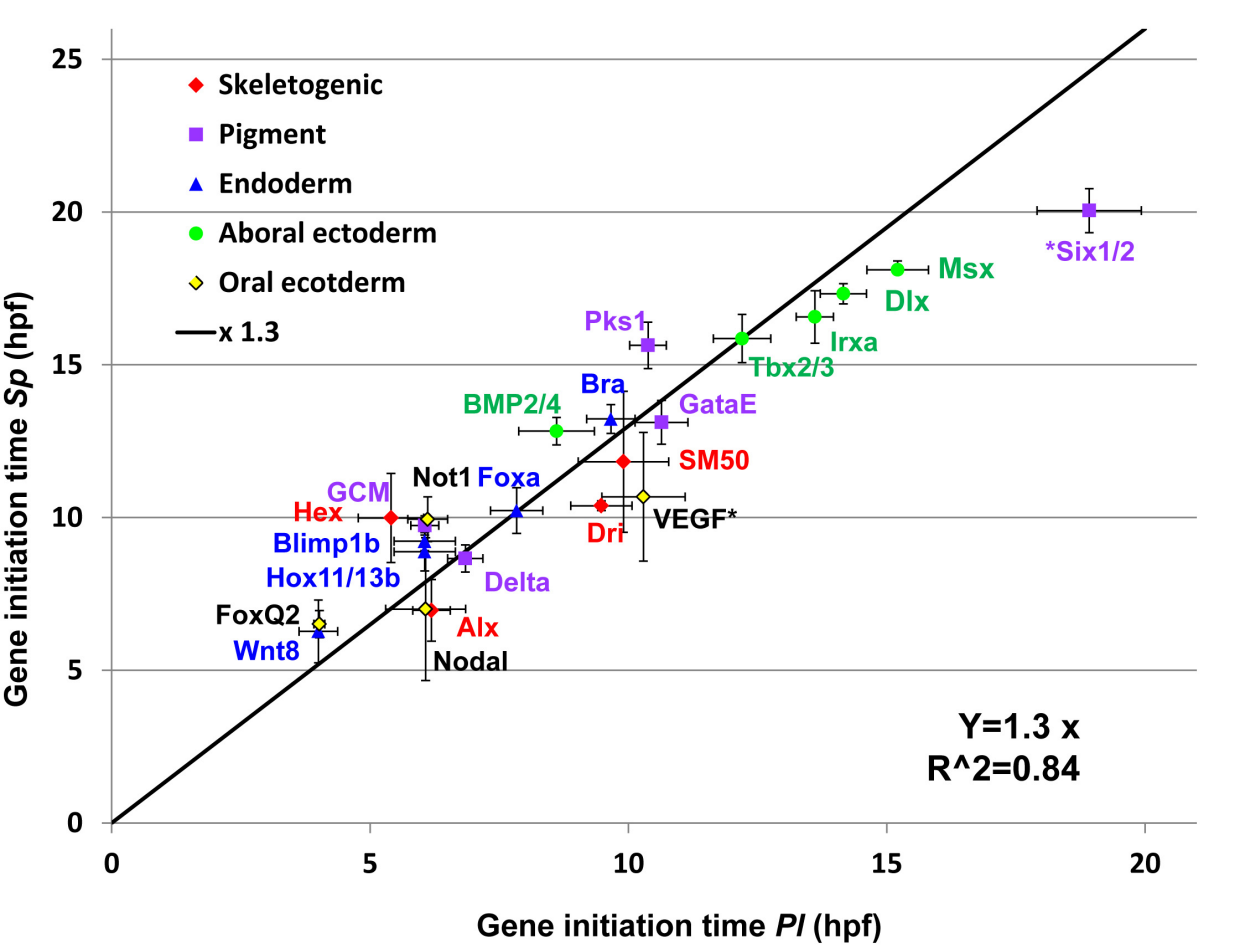
Linear relationship between gene initiation times in *Sp* and *Pl*. Gene initiation times for each species was calculated using the sigmoid fit. Black line is the result of a linear regression of the *Sp-Pl* slope (slope of ×1.3, R^2^ = 0.84, interception was set to zero). The genes *six1/2* and *VEGF* were excluded from the linear regression as their temporal expression profiles indicate changes in their regulation.

We wanted to learn how the molecular developmental rates scale between the two species, across the embryo and over developmental time. Comparison of embryo morphology between the species gives a crude factor of about ×1.3–1.4 between *Pl* and *Sp* developmental rates (Fig 1A), however we wanted to use our data to improve this estimation and base it on measured molecular progression. Since the developmental rates and initiation times are quite repeatable between individuals (Fig 2D and 2E) we used the initiation time to assess the ratio between the species’ developmental rates. We used the sigmoid fit described above log(*mRNA*(*t*)) = *a* − *b*/(1 + exp(*c*(*t* − *t*0)) (Fig 2B) to estimate the initiation time, t0, of each gene, using the average expression profiles presented in Fig 3 [35]. We plotted the estimated initiation times of all *Sp* genes against the initiation times of their orthologues in *Pl* (Fig 4). Gene initiation times in the two species show a high Pearson correlation (0.95) which suggests a linear relationship between the developmental rates of the two species in the developmental window we study. We used linear regression to calculate the ratio between *Sp* and *Pl* developmental rates and obtained factor of ×1.30 (R^2^ = 0.84) between *Pl* and *Sp* developmental rates. This ratio is consistent with the enhanced rate of *Pl* embryo development observed by morphological comparison (Fig 1A) and is similar to the increase in *Sp* developmental rate when cultured at 18°C [3]. The linear relationship between the developmental rates of the two species indicates that despite the observed delays in a few genes in either *Pl* or *Sp*, the developmental progression is quite uniform throughout the embryonic territories and developmental window we study.

#### Temporal scaling and level normalization of *Pl* and *Sp* expression profiles reveals strong interspecies conservation of relative dynamics

The measured expression profiles in both species are highly dynamic within the developmental window we study. Many of the genes have complex spatial expression patterns, and the changes in expression levels correspond to different spatial expression phases. For example, the first peak in *wnt8* expression corresponds to its expression in the skeletogenic mesoderm while the second peak is due to its expansion to the next tier of cells. Thus, the observed changes in expression levels though development reflect the genes’ developmental function through time. If the gene’s developmental function is conserved, we expect to see high conservation of the expression dynamics.

**Fig 5:**
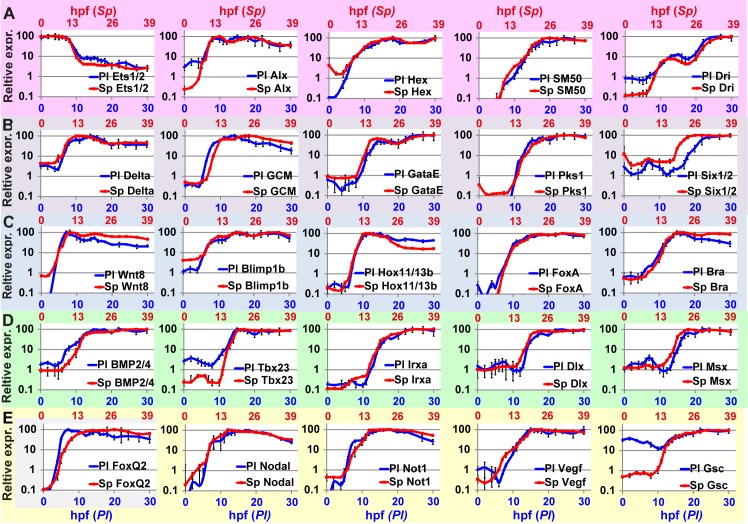
High similarities between *Sp* and *Pl* normalized expression profiles scaled according to developmental rates. Corresponding developmental time points in the two species are provided in S1 Table. **A**, Skeletogenic mesoderm circuit; **B**, aboral non-skeletogenic mesoderm (pigment) circuit; **C**, endoderm circuit; **D**, Aboral ectoderm circuit; **E**, oral ectoderm circuit.

To quantify the similarity between the temporal dynamics of each gene throughout the developmental window, we scaled the developmental rates of the two species and normalized gene expression levels. We used the estimated scaling factor of ×1.3 (Fig 4) to align *Pl* time points with those of *Sp*. We normalized the expression level of each gene by dividing the level at each time point in the maximal mRNA level measured so 100% is the maximal expression in this time interval. In Fig 5, we present the results of this scaling for all the genes studied at the time interval 0–30 hpf in *Pl* and 0–39 hpf in *Sp* (exact time points in each species are provided in S1 Table). The degree of interspecies conservation of relative dynamics is quite striking and indicates tight and highly conserved regulation of the entire kinetic profile throughout development. This alignment allows us to calculate the correlation between gene expression profiles in the two species throughout this developmental window and the resulting *Sp-Pl* correlations are very strong, averaging 0.90 (S3 Fig). In comparison, when we calculate Pearson correlation between random pairs of genes the average correlation decreases to 0.49. Together, these results indicate that the relative changes in expression levels are highly conserved between the species and most likely reflect a highly conserved developmental role.

## Discussion

Embryo development generates similar morphologies despite natural genetic variation and within broad environmental conditions. This flexibility of the developmental program is essential for the survival of the species and keeping a wide genotypic pool adaptable in a changing environment. Understanding the properties of the regulatory control system that underlie cell fate specification is a key to the mechanistic understanding of this developmental stability. Here we studied the reproducibility and conservation of expression dynamics of regulatory circuits in two sea urchin species that shared common ancestor about 40 million years ago and inhabit distinct geographic habitats. Embryo size, cell types and morphologies of these two species are highly similar despite their genomic and geographic distance (Fig 1). Our studies illuminate tight control of gene activation timing within the species (Fig 2) and a striking similarity of relative dynamics revealed by scaling the developmental rates of the two species and normalizing gene expression levels (Fig 5). The regulatory systems that enable this reproducibility and conservation are the underlying mechanisms of morphological similarity amidst genetic and environmental variation.

The high resolution of our studies reveals tight control of initiation times that show lower variation than the variations in maximal mRNA levels between different individuals in the same species (Fig 2). Interestingly, lower variations of initiation time compared to the variation of expression levels were also detected in a comparative study of the developmental transcriptomes of two *Xenopus* species [35]. Previous studies in yeast provide a possible mechanistic explanation of these findings [56,57]. These studies show explicitly that the initiation of gene activation is highly similar for different levels of the activating input once the input level is above a certain threshold for long enough time [56]. On the other hand, once the gene is on, the level of gene expression is highly dependent of the level of the activating input. The molecular explanation for the different behavior of initiation timing and expression level was suggested by the same group several years before [57]. Their measurements and modeling of expression kinetics indicated that the timing of gene initiation is controlled by the slow rate of nucleosome removal from the DNA. Once the nucleosomes are removed, the level of gene expression depends on the affinity of the transcription factor binding sites and the concentration of the activating transcription factor that define the binding site occupancy and the rate of mRNA generation. Thus, the ability to buffer variations in expression level and still tightly control the timing of gene activation, possibly by using nucleosomal positioning as a threshold mechanism, could be a general property of eukaryote gene regulatory networks.

Our interspecies comparison of temporal expression profiles of key regulatory circuits revealed a high conservation of the temporal order of gene activation within the circuits but also some cases of divergence (Fig 3). Integrating the differences in temporal profiles with available perturbation and spatial expression data provides predictions for specific *cis*-regulatory changes within the ectodermal circuits. The highest interspecies conservation of temporal ordering and the timing of gene activation are observed in the endoderm circuit (Figs 2G–2I and 5C). This degree of conservation supports the conservation of both the architecture and the *cis*-regulatory logic of this circuit. The endodermal circuit is one of the most conserved circuits within echinoderms, with a similar architecture detected in the sea star that shared a common ancestor with the sea urchin ~500 mya [58,59]. The mesodermal and ectodermal networks show higher variation of circuit connectivity between the sea urchin and sea star [60–62], emphasizing the strong developmental constraints on the endoderm circuit. The constraints that define this high degree of temporal conservation could be the requirement to initiate gastrulation and the invagination of the gut at the right developmental time. Thus, high-resolution comparison of circuits’ dynamics is a good tool for the prediction of conservation and changes in circuit connectivity when the general circuit structure is known at least in one of the species.

We used gene initiation times measured in the two species to estimate a ×1.3 ratio between the molecular developmental rates in *Pl* and *S*p (Fig 4). Apparently, a major contribution to the accelerated developmental rate in *Pl* is its higher culture temperature compared to the culture temperature of *Sp* (18°C in *Pl* vs. 15°C in *Sp*). A recent study had shown that when *Sp* embryos are cultured in 18°C their developmental rate increases by about ×1.24 fold based on morphological comparison, close to the ratio we obtained [3]. This is in agreement with recent studies in invertebrate and vertebrate embryos that show morphological and molecular scaling with temperature of diverse species [35,63]. A recent morphological comparison of ten *Drosophila* species shows that the rate of embryogenesis scales with temperature within a wide range of temperature (17.5°C-32°C) [63]. A comparative study of two *Xenopu*s species grown in different culture temperature (28°C vs. 22°C) shows that the rate of embryogenesis scales with temperature based on morphology and on the timing of gene activation for most studied genes [35]. Thus, the ability to adapt to different temperatures by scaling the developmental rates without distinct morphological phenotypes is a common property to both vertebrate and invertebrate species.

Our studies reveal remarkable interspecies conservation of expression dynamics when the developmental rates of the two species are scaled and gene expression levels are normalized (Fig 5). This demonstrates an impressive ability of biological developmental systems to tightly control gene activation timing and relative expression dynamics despite genetic and growth conditions differences. This raises the question: Is the observed conservation an outcome of a strong negative selection against genetic changes of regulatory circuits or due to the structure of regulatory circuits that buffers genetic and environmental changes? We tend to support the second option and the ability of the regulatory system to overcome expression noise. This could be achieved by noise filtration mechanisms, *e*.*g*., the threshold activation suggested above, or by the use of network motifs that define different levels of sensitivity to upstream variation. For example, computational studies show that positive feedback circuitry is more efficient than other architectures in buffering noise in the inducing signal while keeping high responsivity to the level of the signal [64,65]. On the other hand, incoherent feedforward motifs can generate consistent response to activating input that depends mostly on fold changes in input and not on noisy absolute protein levels [64,66–68]. Apparently, this flexible design of gene regulatory circuits enables them to conserve similar expression dynamics and specify similar cell types while allowing the species to keep a broad genotypic variance and survive through changing environmental conditions.

## Materials and Methods

### Sea urchin embryo cultures and RNA extraction

Adult sea urchins were supplied from a mariculture facility of the Israel Oceanographic and Limnological research in Eilat. Sea urchin eggs and sperm were obtained by injecting adult sea urchins with 0.5M KCl. Embryos were cultured at 18°C in artificial sea water. Total RNA was extracted using Qiagen mini RNeasy kit from embryos at indicated time points. 1 μg of total RNA from each time point of each three independent biological replicates was used to generate cDNA using BioRad i-script kit and subsequently used for QPCR.

### QPCR

#### Protocols

QPCR reactions were executed in 384-well plates using 384CFX-real time machine (BioRad). Each reaction was run in experimental triplicate and biological triplicate, hence leading to at least nine measurements per gene for each time point. Every reaction contained 10 μl SYBR Green mix from BioRad including 3 μM forward and reverse gene pecific primers and 2.5 μl of cDNA (diluted 1:100 for each assay). Thermal cycling parameters were 95°C for 3 min (one cycle) and then 95°C for 10 s, 55°C for 10 s, and 72°C for 30 s (40 cycles), followed by a denaturation step to verify the amplification of a single product.

#### Primer design and efficiency

Sequences of *Pl* genes were retrieved by blastn searches of the transcriptome databases available by permission on the Octupus web portal (http://octopus.obs-vlfr.fr/) using as templates the annotated sea urchin *Sp* mRNA sequences retrieved from the sea urchin transcriptome web page (http://www.spbase.org:3838/quantdev/). Based on these sequences we designed QPCR primers for each gene using Primer3 web site (http://primer3.ut.ee/). We wanted to capture transcription initiation time and therefore the primers were designed for the most 5' end 500 bp of each gene, within the ORF. The size of the amplicons was 120–150 bp long. Primer sequences are available in supporting S2 Table. All primer pairs were initially validated by regular PCR amplification and their amplification efficiencies were subsequently determined by standard curve analyses carried out using 4-fold serial dilutions of appropriate cDNA samples. Only primers with an amplification efficiency ranging from 1.85 to 2.03 (hence 89–103%) were used for further analyses.

#### Quantification of gene expression

To quantify the relative levels of mRNA per sample in *Pl* we inserted a known number of GFP cDNA molecules to each sample that includes cDNA transcribed from 1.25 ng of extracted total RNA of *Pl* at different time points. The calculation of gene prevalence compared to GFP was performed using the formula, GFP × 1.9^(*CtGFP*−*Ctgene*)^, with a constant coefficient efficiency factor, 1.9, corresponding to the average value of all primers set. In our experiments we used either 2.6×10^8^ or 1.1×10^7^ GFP molecules and obtained similar results. The measured value of total RNA of *Sp* embryo is about 2.8 ng. Considering the efficiency of the extraction procedure and RNA instability we estimated that about 1.25 ng of total RNA extracted by RNeasy kit is equivalent to RNA of roughly one embryo, so the mRNA levels we present are a good estimate for number of mRNA copies per embryo. Technical error was measured as $\sqrt{{SEM}_{GFP}^{2}+{SEM}_{Gene}^{2}}$, where SEM is the standard error of the QPCR measurement. Percent of standard deviation is calculated as standard deviation of a quantity (either maximal mRNA level or initiation time) measured at the three biological repeats divided by the average quantity measured. To generate Fig 5, we normalized each averaged time course by dividing the measured level at each time point by the maximum level of this gene during the time window of the experiment.

### Data analysis

Initiation times, t0, for both Fig 2D and Fig 4 were estimated by the use of the sigmoid function: Log(*mRNA*(*t*)) = *a* − *b*/(1 + exp(*c*(*t* − *t*0)) as in Yanai *et el*, 2011 [35]. The sigmoid was fit using Matlab’s Curve Fitting Toolbox, using the nonlinear least-squares method. For all genes R^2^>0.94, except from PlDlx repeat #3 that had R^2^ = 0.88. To calculate Pearson correlation between *Sp* and *Pl* time course, we first scaled the developmental rates in the two species using a factor of ×1.3 between *Pl* and *Sp*. The exact time points we compared are given in S1 Table. Then we calculated Pearson correlation between the averaged expression levels in *Sp* and *Pl* using the CORREL function in excel.

## Supporting Information

S1 Fig Typical network circuitries and the dependence of circuit dynamics in *cis*-regulatory logic. **A-C**, illustration of the dependence of temporal expression profile of a gene in the temporal expression profile of its inputs and the logic applied on the input by the gene *cis*-regulatory elements. The simulations are based on the mathematical model presented in **[9] A**, Gene C is activated by two inputs, transcription factors A and B. **B**, the expression of A preceded B expression and thus the initiation time of gene C depends on the logic applied on the inputs. If both inputs are necessary for C activation, (AND logic), gene C will turn on only after B is on. **C,** if both inputs are sufficient to activate C, (additive, OR logic), gene C will turn on immediately after A onset. Thus, evolutionary changes of the logic applied on input can induce temporal changes in gene activation. **D-F,** typical network motifs, **D**, positive feedback; **E**, Inter-cellular positive feedback (community effect); **F**, Positive feedforward loop.
(TIF)

S2 Fig Temporal expression profiles in *Pl*. Relative mRNA levels were calculated relatively to GFP known quantity at each point for each biological repeat (See material and methods for experimental details). Different biological repeats are indicated in different colors.
(TIF)

S3 Fig *Sp-Pl* Pearson correlation of temporal expression profiles. (TIF)

S1 Table Equivalent time points in *Pl* and *Sp* used to scale the developmental rates in the two species and to calculate the correlation of temporal profiles. (DOCX)

S2 Table List of QPCR primers that were used in this work based on sequences retrieved by blastN searches of the transcriptome databases available by permission on the Octupus web portal (http://octopus.obs-vlfr.fr/) using as templates the annotated sea urchin *S*. *purpuratus* mRNA sequences retrieved from the sea urchin transcriptome web page (http://www.spbase.org:3838/quantdev/). (DOCX)

S1 Dataset QPCR measurements of 25 *Pl* genes at the three biological repeats, averages and standard deviations. (XLSX)
