# Supplementary figures and images for: Comparative Study of Regulatory Circuits in Two Sea Urchin Species Reveals Tight Control of Timing and High Conservation of Expression Dynamics
Source: PLoS Genet. 2015 Jul 31;11(7):e1005435. doi: 10.1371/journal.pgen.1005435 (PMC4521883; doi:10.1371/journal.pgen.1005435)

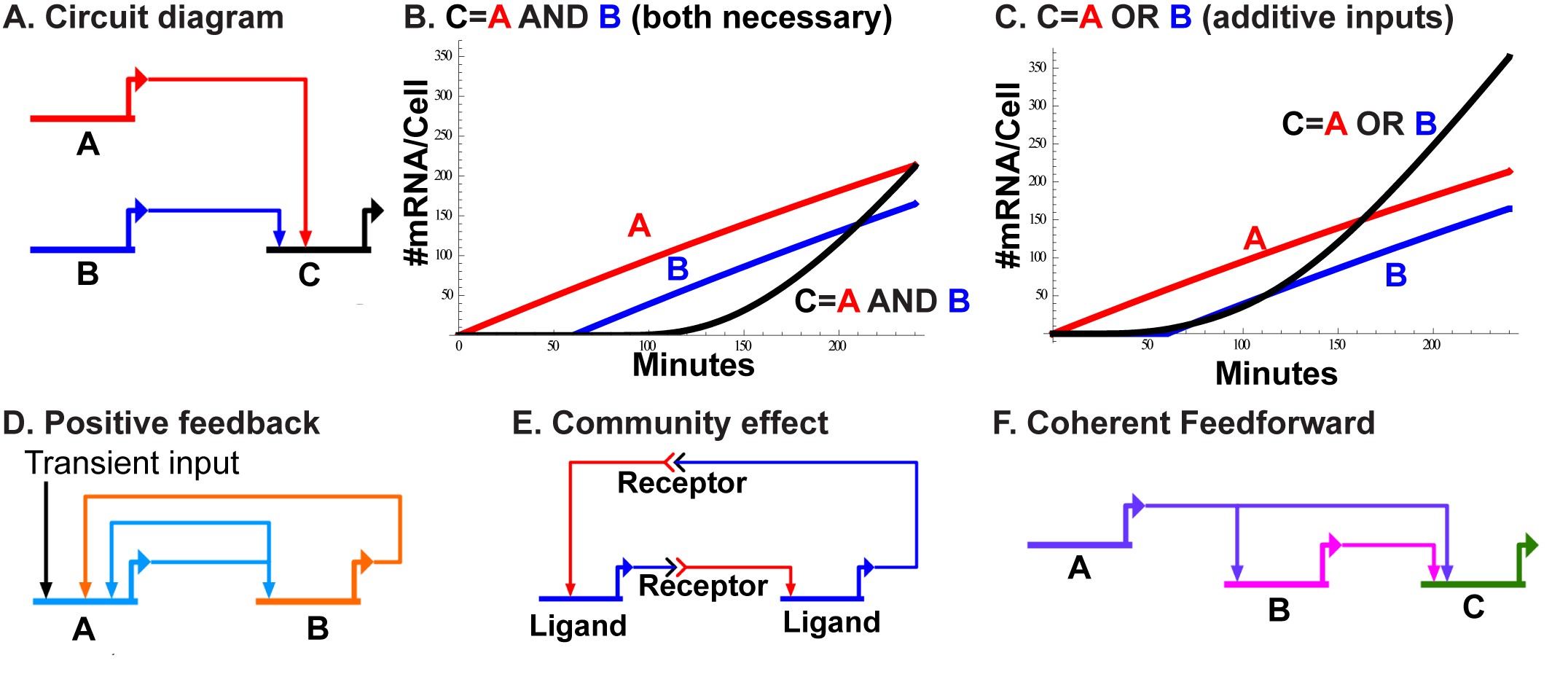

Supplement: S1 Fig — A-C, illustration of the dependence of temporal expression profile of a gene in the temporal expression profile of its inputs and the logic applied on the input by the gene cis-regulatory elements. The simulations are based on the mathematical model presented in [9] A, Gene C is activated by two inputs, transcription factors A and B. B, the expression of A preceded B expression and thus the initiation time of gene C depends on the logic applied on the inputs. If both inputs are necessary for C activation, (AND logic), gene C will turn on only after B is on. C, if both inputs are sufficient to activate C, (additive, OR logic), gene C will turn on immediately after A onset. Thus, evolutionary changes of the logic applied on input can induce temporal changes in gene activation. D-F, typical network motifs, D, positive feedback; E, Inter-cellular positive feedback (community effect); F, Positive feedforward loop. (TIF) [file pgen.1005435.s001.tif]

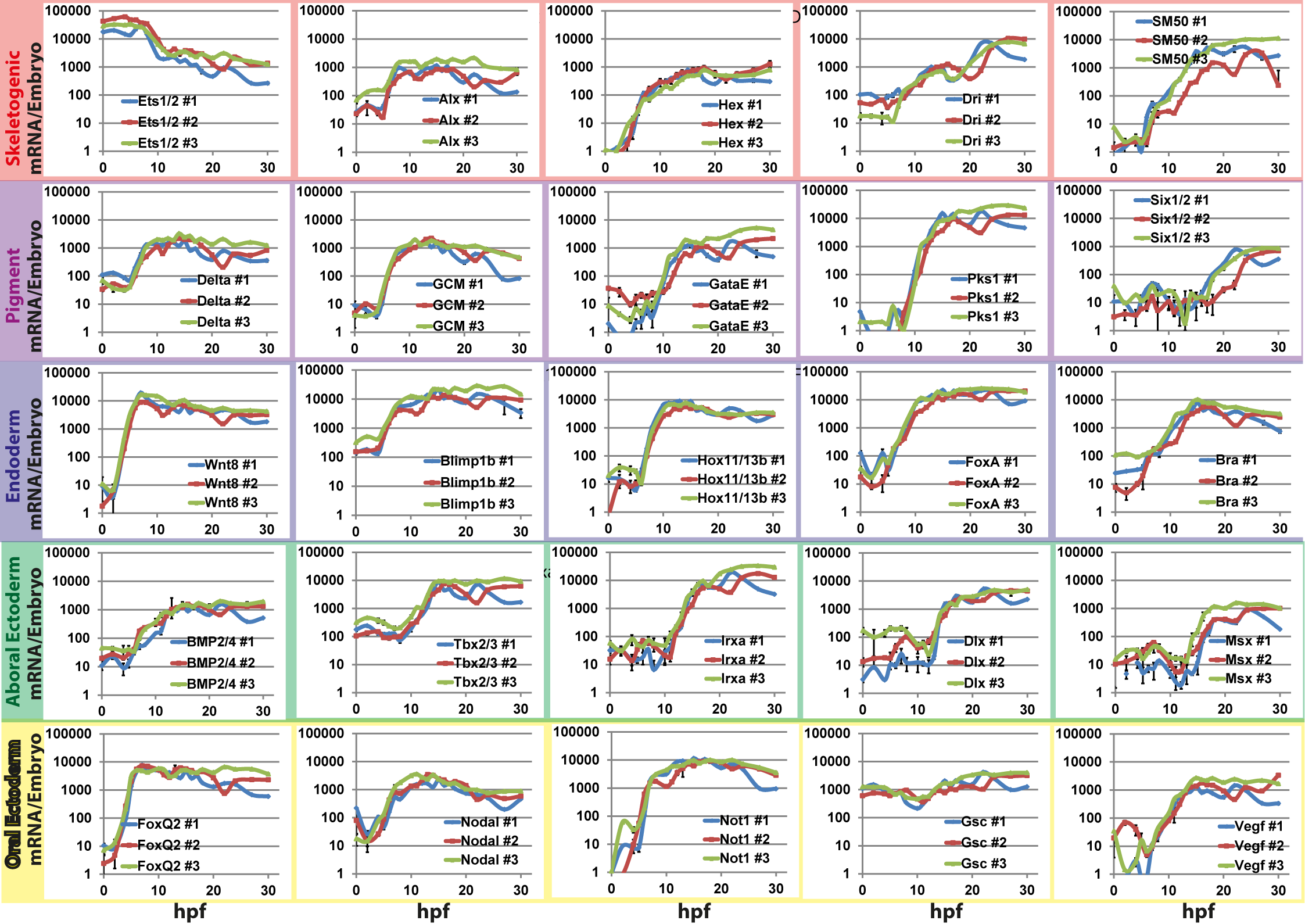

Supplement: S2 Fig — Relative mRNA levels were calculated relatively to GFP known quantity at each point for each biological repeat (See material and methods for experimental details). Different biological repeats are indicated in different colors. (TIF) [file pgen.1005435.s002.tif]

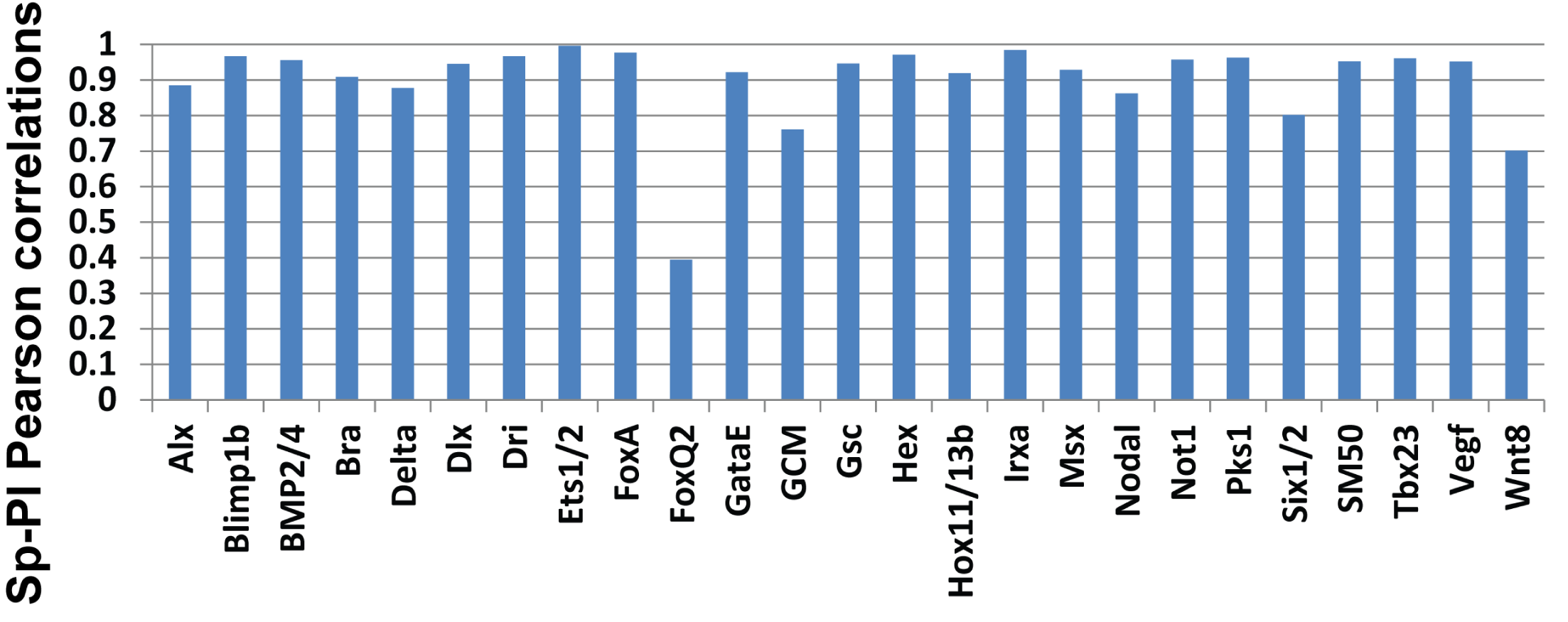

Supplement: S3 Fig — (TIF) [file pgen.1005435.s003.tif]
