# Supplementary material for: Comparative Study of Regulatory Circuits in Two Sea Urchin Species Reveals Tight Control of Timing and High Conservation of Expression Dynamics
Source: PLoS Genet. 2015 Jul 31;11(7):e1005435. doi: 10.1371/journal.pgen.1005435 (PMC4521883; doi:10.1371/journal.pgen.1005435)
Supplement: S1 Table — (DOCX) [file pgen.1005435.s004.docx]

| **Sp hpf** | **Pl hpf** |
| --- | --- |
| 0 | 0 |
| 3 | 2 |
| 5 | 4 |
| 7 | 5 |
| 8 | 6 |
| 9 | 7 |
| 10 | 8 |
| 13 | 10 |
| 14 | 11 |
| 16 | 12 |
| 17 | 13 |
| 18 | 14 |
| 20 | 15 |
| 21 | 16 |
| 22 | 17 |
| 24 | 18 |
| 26 | 20 |
| 29 | 22 |
| 31 | 24 |
| 35 | 27 |
| 39 | 30 |

Table S 1
