# Supplementary material for: Comparative Study of Regulatory Circuits in Two Sea Urchin Species Reveals Tight Control of Timing and High Conservation of Expression Dynamics
Source: PLoS Genet. 2015 Jul 31;11(7):e1005435. doi: 10.1371/journal.pgen.1005435 (PMC4521883; doi:10.1371/journal.pgen.1005435)
Supplement: S2 Table — (DOCX) [file pgen.1005435.s005.docx]

| **Gene name** | **QPCR primer F** | **QPCR primer R** |
| --- | --- | --- |
| **Alx1** | aatttgcagaacctccgacg | tcattgaggagtggttggct |
| **Blimp1b** | AGAAGAGGTGCCAGCAGATG | CTACCAGGTTCTGCTCTGCC |
| **BMP2/4** | GTACCGGTCGCATACA CAAG | TGTGTCTGTGCTGCTCTGTA |
| **Bra** | TGAAAGTCAGGCTGGAGGAC | AGTACATGGAGTTCGGGTCG |
| **Delta** | GCACATTCGCTTTCCGTAAT | AGACCCTGTGCTGCTTTCAT |
| **Dlx** | GGGCATCCTCCAATTTATGA | GCAAGGTATTGGGTCTGGTG |
| **Dri** | TGGAAATAGACGAGAGGGGC | GTGGTATCATGGTGGGTGGA |
| **Ets1/2** | CCAACATGCATCTGGACGAG | CCGGACCGCCAATCATTATG |
| **FoxA** | caggtatgggaagcatggga | gcgtatctcatcgacatggc |
| **FoxQ2** | CCACCATCAACCAAGAAACC | TCCAGCTTCTTTCGTTGTCC |
| **Gata4/5/6** | CACCCCTGATACGACCTACC | GTGATGCATGCCTGAGAAGG |
| **GCM** | ATTTTCGACATCCCTGCTGC | CTTGAAGTACCTGCGGCATC |
| **GFP** | AGGGCTATGTGCAGGAGAGA | CTTGTGGCCGAGAATGTTTC |
| **Gsc** | CAGTCCCTCATCACCTCCAT | AGCCGGTGTAAGGGTTGTAG |
| **Hex** | TGAACCACCCTACTCCACTG | GCCGCTCTATTTTGTCCGAG |
| **hox11/13b** | TTGCGACGTTCACAACAACT | GTGAGATCGAGAGCCTGTGA |
| **Irxa** | CTACCCACCGCACTCTACTC | GCGTTCCCTTCAAATCAGCA |
| **Msx** | TCCTGTAACGGCCATCTCTC | CTGTTGGGTGGTGTTTCGAC |
| **Nodal** | GCATTGAACTCCGCTCCAAA | TCGCCGTCCTCACTAGAATC |
| **Not1** | GTCTGAAACGCCTTCGATGT | GGCGAGGTAGAGTCGTTCAC |
| **PKS1** | TTGTAGGCATTGGTACCCGT | TTGCCTTGTCTGATCCTCGT |
| **Six1/2** | TTGGACGATTTCTCTGGTCA | GGTTATGCGGCGAGAAGTTA |
| **SM50** | GGACCTGCTAAAATGGGCTC | TCCATCCAAGCCAGATCTCA |
| **Tbx2/3** | TATTACCACCTCGCCTCAGC | CCTGGATGTTCGCCGAATTT |
| **Vegf** | GCTCATGGTTCTCTCGAAGG | CCCGCTGAGATAACATTGGT |
| **Wnt8** | AAGTGTCATGGCGTCTCTGG | ATGAGCTTGCCACTGACGAA |

**Table S2. QPCR primer sequences.**
